# LVAD as a Bridge to Remission from Advanced Heart Failure: Current Data and Opportunities for Improvement

**DOI:** 10.3390/jcm11123542

**Published:** 2022-06-20

**Authors:** Christos P. Kyriakopoulos, Chris J. Kapelios, Elizabeth L. Stauder, Iosif Taleb, Rana Hamouche, Konstantinos Sideris, Antigone G. Koliopoulou, Michael J. Bonios, Stavros G. Drakos

**Affiliations:** 1 Divisions of Cardiovascular Medicine and Cardiothoracic Surgery, University of Utah Health & School of Medicine, Salt Lake City, UT 84132, USA; christos.kyriakopoulos@utah.edu (C.P.K.); chriskapel@hotmail.com (C.J.K.); lizzy.stauder@hsc.utah.edu (E.L.S.); joseph.taleb@hsc.utah.edu (I.T.); konstantinos.sideris@hsc.utah.edu (K.S.); koliopoulou.a@gmail.com (A.G.K.); bo_mic@yahoo.com (M.J.B.); 2 Nora Eccles Harrison Cardiovascular Research and Training Institute, University of Utah, Salt Lake City, UT 84112, USA; rana.hamouche@utah.edu; 3 Divisions of Cardiology & Cardiothoracic Surgery, Onassis Cardiac Surgery Center, 17674 Athens, Greece

**Keywords:** heart failure, mechanical circulatory support, left ventricular assist device, reverse remodeling, myocardial recovery

## Abstract

Left ventricular assist devices (LVADs) are an established treatment modality for advanced heart failure (HF). It has been shown that through volume and pressure unloading they can lead to significant functional and structural cardiac improvement, allowing LVAD support withdrawal in a subset of patients. In the first part of this review, we discuss the historical background, current evidence on the incidence and assessment of LVAD-mediated cardiac recovery, and out-comes including quality of life after LVAD support withdrawal. In the second part, we discuss current and future opportunities to promote LVAD-mediated reverse remodeling and improve our pathophysiological understanding of HF and recovery for the benefit of the greater HF population.

## 1. Introduction

Heart failure (HF) is characterized by a pathologic process known as remodeling that involves systolic and diastolic impairment, progressive ventricular dilation, and an increase in intracardiac pressures. The process of remodeling is associated with adverse cellular, structural, and functional myocardial changes, that have long been deemed progressive and unidirectional. Clinical experience has shown that the process of remodeling can be delayed or even reversed, either spontaneously in the setting of acute cardiac injury (e.g., acute myocarditis, stress-induced cardiomyopathy etc.), or it can be facilitated through guideline-directed HF therapy including cardiac resynchronization therapy in chronic HF [1,2,3]. Mechanical circulatory support (MCS) with left ventricular assist devices (LVADs) is an established treatment modality for patients with advanced disease and besides its role in supporting systemic circulation by augmenting cardiac output, it provides significant volume and pressure unloading, creating a favorable environment for the reversal of the structural and functional alterations of the failing heart, a process known as reverse remodeling. It has been repeatedly shown that a subset of advanced HF patients can significantly improve their cardiac structure and function while on durable MCS, to the point where withdrawal of the LVAD support can be considered [4,5,6,7,8,9,10,11,12]. In light of these findings, the concept of HF irreversibility has been refuted and the notion that severe HF requiring durable LVAD support indicates irreversible end-stage disease has been revised.

## 2. Historical Perspective

In the recent past, left ventricular remodeling was widely considered irreversible, especially in patients with advanced disease. Besides evidence that the remodeling process could be attenuated with early implementation of angiotensin-converting enzyme inhibitors (ACEi) after a myocardial infarction, the concept that a profoundly dilated ventricle in end-stage HF could revert to a significantly improved phenotype had not been reported [13,14]. The first challenges to this notion were largely driven by the observation that advanced HF patients can improve their cardiac function after MCS. In 1994, Frazier et al. were the first to describe cardiac improvement in a series of 18 patients supported with either a pneumatic or vented electric HeartMate^®^ (Thoratec Corporation, Pleasanton, CA, USA) device [15,16]. The investigators observed that LVAD support resulted in a significant reduction in the cardiothoracic ratio and left ventricular end-diastolic diameter (LVEDD), and in an improvement in the left ventricular ejection fraction (LVEF) from the baseline. Improved hemodynamics, including a decreased pulmonary capillary wedge pressure (PCWP) and an enhanced cardiac index were also noted in these patients, while histologic examinations demonstrated a reduction in the mean area of myocytes. Close to that time, Levin et al. measured the end-diastolic pressure–volume relationship and examined the cardiac tissue at the time of heart transplantation in patients treated with medical therapy compared to patients bridged with LVAD support [17]. They observed that the LVAD support was associated with a regression of the cellular hypertrophy and a shift of the end-diastolic pressure–volume relationship towards normal values, suggesting a reversal of the remodeling process.

As these findings were often accompanied by a significant recovery of the underlying cardiac function, the first cases where the left ventricular functional improvement was sufficient to allow for LVAD removal (cardiac recovery or a “remission” of HF) were reported [10,11,18]. The first cases of LVAD explantation were performed without using standardized criteria to assess the left ventricular recovery; however, in the following years a more standardized approach was followed and criteria for device weaning were introduced and implemented [4,7,9,19,20,21]. The first anecdotal experiences were followed by reports showing high rates of cardiac recovery and subsequent LVAD explantation, by combining LVAD support with adjuvant reverse remodeling drug therapy, alongside regular testing of the underlying cardiac function [5,6,22,23]. Henceforth, an increasing number of institutions adopted approaches of facilitating and testing for recovery and potential LVAD explantation, leading to the RESTAGE-HF (Remission from Stage D Heart Failure) multicenter study [4]. In this prospective trial, 19 out of the 40 (47.5%) selected chronic advanced HF patients undergoing LVAD support combined with a standardized pharmacologic and cardiac function monitoring protocol, markedly improved their left ventricular structure and function, and had the device explanted. Several cohorts of patients now exist who have had their device removed and have had sustained recovery for many years [4,20,24,25,26]. These patients have been able to return to a normal lifestyle without requiring heart transplantation, thereby enabling the allocation of donor hearts to other individuals in need of this precious resource.

## 3. Incidence, Magnitude and Time Course of Cardiac Recovery

After the initial anecdotal experiences of cardiac recovery in the 1990s, several subsequent reports investigated the phenomenon in a series of LVAD-explanted patients, trying to shed light on the incidence and long-term outcomes.

One of the first reports of marked left ventricular structural and functional improvement on LVAD support and subsequent device explantation was in a small series of five patients with advanced HF and underlying non-ischemic cardiomyopathy (idiopathic dilated cardiomyopathy in three, and postpartum cardiomyopathy in two of the patients) [10]. In three of these patients, the LVAD was removed electively after the recovery of cardiac function, while in the remaining two, it was removed because of device malfunction. While 1 patient died of a non-cardiac cause 10 days after LVAD removal, the other 4 patients remained alive and well 35, 33, 14, and 2 months after LVAD removal, respectively.

The multicenter Thoratec Registry provided one of the largest patient cohorts and included a total of 281 patients with underlying non-ischemic cardiomyopathy [9]. A total of 22 out of 281 (8.1%) patients underwent LVAD explantation, with 17 patients remaining alive, 16 in New York Heart Association (NYHA) class I, and 1 in NYHA class II, after an average follow-up of 3.2 years (1.2–10 years). In another large series, Mancini et al. retrospectively reviewed 111 patients receiving an LVAD as a bridge to transplantation [18]. Only 5 of the 111 patients (4.5% overall, and 9% of patients with non-ischemic HF etiology) had substantial cardiac recovery and were deemed appropriate for device explantation. Notably, just 1 patient remained alive with sustained left ventricular improvement after 15 months of follow-up.

Subsequent studies by the Berlin group reported higher rates of LVAD removal and more sustained left ventricular recovery. Their initial report showed that all 5 patients who underwent explantation exhibited a preserved cardiac function in the following 51 to 592 days [21]. In succeeding reports by 2005, 32 out of 131 (24%) patients with non-ischemic HF underwent device explantation and exhibited a five-year survival rate of 78.3 ± 8.1% [20]. The explanted patients were free from HF symptoms recurrence at a rate of 69.4% and 58.2% at three and five years, respectively. By 2008, 81 patients were weaned from left, right, or biventricular assist devices that had been implanted for end-stage HF in the same center [27]. When only patients with non-ischemic cardiomyopathy were analyzed and after excluding patients with proven myocarditis, it was found that 35 out of 188 (18.5%) patients underwent device explantation. Thirty patients had the device explanted electively, while in another five the decision was precipitated by pump-related complications. In eight of the electively weaned patients, the LVEF had not normalized (30% to 44%) and the LVEDD was between 56 to 60 mm. Nonetheless, the overall 5- and 10-year survival rates following LVAD explantation, including survival after heart transplantation for patients with HF recurrence, were 79.1 ± 7.1% and 75.3 ± 7.7%, respectively. A major finding through these series was that patients with long-term weaning stability had a shorter duration of HF, were younger, and required MCS for a shorter interval [7,28,29].

The LVAD Working group presented a prospective multi-center study of 67 patients who received an LVAD for refractory HF across eight centers in the US [30]. Thirty-seven patients had an underlying non-ischemic, and thirty an ischemic cardiomyopathy, while all patients underwent implantation of the HeartMate^®^ XVE LVAD (Thoratec Corporation, Pleasanton, CA, USA). On an echocardiographic follow-up, the LVEF increased from 17 ± 7% before LVAD implantation to 34 ± 12% (*p* < 0.001), the LVEDD decreased from 7.1 ± 1.2 cm to 5.1 ± 1.1 cm (*p* < 0.001), and the left ventricular mass decreased from 320 ± 113 g to 194 ± 79 g (*p* < 0.001). Peak oxygen consumption (pVO_2_) improved while on LVAD support (13.7 ± 4.2 mL/kg/min at 30 days vs. 18.9 ± 5.5 mL/kg/min at 120 days; *p* < 0.001). Overall, six (9%) patients were weaned from their LVAD due to cardiac recovery.

The Utah Cardiac Recovery Program reported on 154 consecutive, prospectively enrolled patients with chronic advanced HF receiving a continuous-flow LVAD, after excluding patients with an acute HF etiology [31]. They reported that 21% of patients with an underlying non-ischemic and 5% of those with an ischemic cardiomyopathy achieved an LVEF ≥ 40% after at least six months on LVAD support, while the end-diastolic and end-systolic volumes were significantly and similarly improved in both cohorts.

Recently, the results of the multicenter RESTAGE-HF trial were published [4]. Forty patients with chronic advanced HF receiving the HeartMate II™ LVAD (Thoratec Corporation, Pleasanton, CA, USA) were enrolled across six US centers. An underlying non-ischemic cardiomyopathy, LVEF < 25% with cardiomegaly, age < 60 years, and a duration of HF < 5 years were the inclusion criteria. The LVAD speed was optimized, a HF pharmacological regimen was implemented, and regular echocardiograms were performed at a reduced LVAD speed to test the underlying cardiac function. Prior to LVAD implantation, the LVEF was 14.5 ± 5.3% and the LVEDD was 7.33 ± 0.89 cm. Four enrolled patients did not abide by the protocol due to medical complications unrelated to the study procedures. Overall, 40% of all the enrolled (16/40) patients achieved the primary endpoint of a marked left ventricular structural and functional improvement, a subsequent LVAD explantation within 18 months, and sustained remission from HF at 12 months. Half (18/36) of the patients receiving the protocol were explanted within 18 months (pre-explant LVEF 57 ± 8%; LVEDD 4.81 ± 0.58 cm; LV end-systolic diameter 3.53 ± 0.51 cm; PCWP 8.1 ± 3.1 mmHg; pulmonary artery saturation 63.6 ± 6.8% at 6000 rpm), while overall, 19 patients were explanted (19/36, 52.3% of those receiving the protocol). A post-explantation survival rate, free from reimplantation of an LVAD or heart transplantation, was 90% at one year, and 77% at two and three years. The investigators concluded that a strategy of LVAD support combined with a standardized pharmacologic and cardiac function monitoring protocol resulted in high rates of LVAD explantation and was feasible and reproducible with explantations taking place in all the participating sites.

A recent analysis of 358 consecutive patients with HF with a reduced LVEF (where patients with acute HF etiologies and less than three months of post-LVAD echocardiographic follow-up were excluded by the study design) receiving a continuous-flow LVAD across four US institutions, showed that 34 (10%) patients had an LVEF ≥ 40% and an LVEDD ≤ 6.0 cm at the last available echocardiographic follow-up timepoint within one year of LVAD support [32]. An additional 112 (31%) patients had an absolute LVEF improvement of ≥5% post-MCS (A median LVEF increase of 9% with a range of 6–14%), with the authors suggesting that such patients may benefit from further mechanical unloading or titration of a guideline-directed HF medical therapy to further improve their cardiac structure and function.

The overall rate of cardiac recovery leading to device weaning in the Interagency Registry for Mechanically Assisted Circulatory Support (INERMACS) database has consistently been low, occurring in less than 5% of the implant cases by five years [33]. Topkara et al., however, studied 13,454 patients implanted with a continuous flow LVAD from 2006 to 2015 using the INTERMACS registry [34]. In this line of investigation, cardiac recovery during LVAD support was defined as complete if the device explantation was performed, or as partial if the patient demonstrated a substantial improvement of the left ventricular systolic function (LVEF > 40%) at any follow-up echocardiographic assessment, yet not achieving the device explantation clinical endpoint. Out of 8805 patients with an LVEF < 30% at the time of device implantation, 761 (8.6%) achieved a partial cardiac recovery, with 406 (4.6%) patients reaching an LVEF in the range of 40–50% and 355 (4.0%) patients an LVEF greater than 50%. Again, by using the INTERMACS registry, the Utah group found that out of 15,631 LVAD patients, approximately 13% either underwent device explantation for cardiac recovery or achieved a follow-up LVEF > 40% [35]. This apparently high discrepancy between the patients achieving marked left ventricular structural and functional improvement, and those who eventually had their LVAD explanted, was not unanticipated. This reflects the complexity of decision-making in favor of LVAD weaning, a decision that is affected by various factors pertaining, among others, to the physician’s expertise, institutional experience, and patient and physician perspectives and goals. Probably the most important factor affecting that decision is the fact that the alternative option of heart transplantation is the gold standard therapy for end-stage HF compared to LVAD explantation, which is, in reality, still under clinical investigation/research.

The incidence of LVAD-mediated cardiac recovery is highly variable in the literature, likely representing a variability in study design, patient selection criteria and definition of cardiac recovery, including the acceptable thresholds of left ventricular improvement to allow for device weaning. The results of key clinical outcome studies investigating cardiac functional and structural improvement following long-term MCS therapy in a prospective way are summarized in Table 1 [4,5,6,7,22,23,27,30,31,36,37,38,39,40].

**Figure 1:**
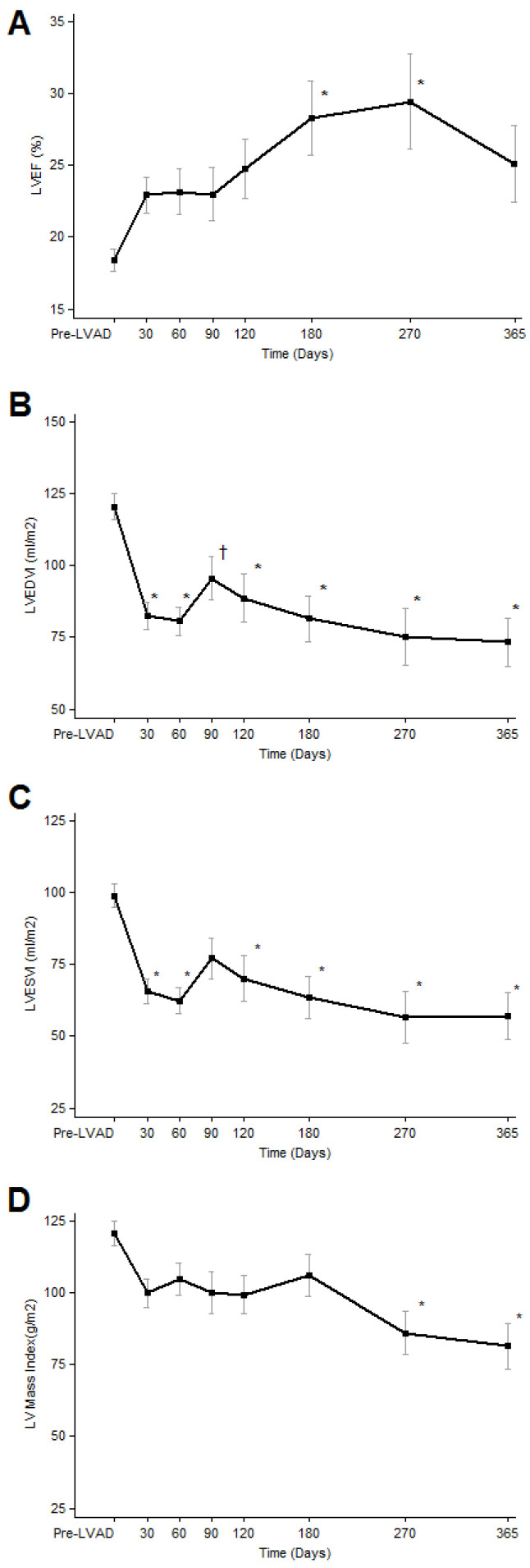
Serial echocardiographic changes in LVAD-supported patients. (**A**). LVEF; left ventricular ejection fraction, (**B**). LVEDVI: left ventricular end diastolic volume index, (**C**). LVESVI; left ventricular end systolic volume index, (**D**). LV (left ventricular) Mass index. Data are presented as means and confidence intervals, * *p* < 0.01 vs. Pre LVAD; † *p* < 0.05 vs. Pre LVAD. ***(Figure reproduced from Drakos SG et al. Magnitude and time course of changes induced by continuous-flow left ventricular assist device unloading in chronic heart failure: insights into cardiac recovery. J Am Coll Cardiol. 2013;61(19):1985–1994) [8]***.

The time course and magnitude of improvement in cardiac structure and function after LVAD placement was prospectively evaluated in a study of 80 consecutive patients with chronic HF, due to both ischemic and non-ischemic cardiomyopathy, who underwent implantation of a continuous-flow device from 2008 to 2011 [8]. The cardiac recovery was assessed on the basis of systolic and diastolic echocardiographic improvement, which was sustained during echocardiograms performed at a reduced pump speed. The serial echocardiographic assessment took place at regular intervals for one year. After six months of LVAD unloading, 34% of the patients had a relative LVEF increase above 50% (compared with the pre-implantation values) and 19% of the patients achieved an LVEF ≥ 40%, irrespective of the underlying HF etiology. This improvement in systolic function was seen as early as 30 days after device implantation with the greatest magnitude of improvement achieved by six months and persisting over the one-year follow-up period. The left ventricular diastolic function parameters improved as early as 30 days and persisted over time. The left ventricular end-diastolic and end-systolic volumes decreased significantly as early as 30 days post-MCS (113 vs. 77 mL/m^2^; *p* < 0.01, and 92 vs. 60 mL/m^2^; *p* < 0.01, respectively). The left ventricular mass also decreased as early as 30 days after circulatory support initiation (114 vs. 95 g/m^2^) and continued to decrease over the one-year follow-up. Importantly, it did not reach values below the normal reference range, suggesting there was no atrophic remodeling after prolonged LVAD support. The above findings are depicted in Figure 1.

**Figure 2:**
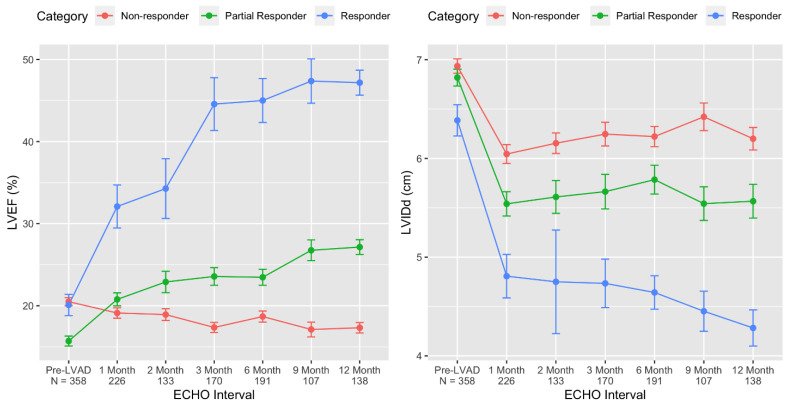
Time course of cardiac structural and functional improvement on LVAD support based on responder stage. By comparing the baseline and last follow-up echocardiogram, changes in the left ventricular ejection fraction (LVEF) and left ventricular internal dimension at end-diastole (LVIDd) were used to categorize LVAD patients into 3 distinct groups: responders (blue), partial responders (green), and non-responders (red). Using serial echocardiography, the change in function (LVEF) and structure (LVIDd) after LVAD implant are depicted by responder category. ***(Figure reproduced from Shah P et al. Framework to Classify Reverse Cardiac Remodeling with Mechanical Circulatory Support: The Utah-Inova Stages. Circ Heart Fail. 2021 May;14(5):e007991) [32].***

Subsequent studies confirmed that improvements in the left ventricular function (LVEF) trailed temporally behind the improvements in left ventricular structure (LVEDD) (Figure 2) [31,32,35], but although functional improvement can be observed at any time after LVAD implantation, most patients eventually achieving an LVEF ≥ 40% exhibit a significant functional improvement on durable MCS by 6–9 months [31,32,35].

## 4. Assessment of LVAD-Mediated Cardiac Recovery

The serial monitoring of cardiac size, geometry, and function following LVAD implantation is of paramount importance to identify the patients with sufficient cardiac improvement to allow for device weaning and a safe, accurate and reproducible protocol for monitoring cardiac recovery is required. Patients are studied under conditions of limited or discontinued LVAD support to examine the underlying cardiac function; however, relevant guidelines do not exist, and as such, the current practice is largely driven by local institutional criteria, case reports and case series, with the available literature consisting mostly of expert opinions. At present, evaluation of cardiac morphology and function by echocardiography and right heart catheterization (RHC) are the mainstays of cardiac recovery assessment and weaning decision-making in LVAD recipients, while additional information obtained through cardiopulmonary exercise testing (CPET) provides supplementary guidance [41,42]. Table 2 provides an overview of commonly used criteria for evaluating cardiac structural and functional improvement and potential MCS weaning in LVAD-supported patients [43].

### 4.1. Echocardiography

The echocardiographic assessment of left ventricular improvement is based on data obtained at rest and during a repeated short-term, discontinued LVAD support or reduced LVAD speed, while the patient is optimally anticoagulated [4,5,7,8,27,41,44,45,46,47]. Ideally, such testing at low LVAD speeds should be repeated regularly to assess the underlying cardiac function, to allow for optimization of the LVAD unloading, and to guide the pharmacologic management of patients to promote reverse cardiac remodeling. Pulsatile-flow LVADs allow for the assessment of native heart function during complete pump stops, as the inflow and outflow valves prevent regurgitation of blood from the aorta to the left ventricle during device deactivation [20,21,27,41,47,48]. However, in the case of continuous-flow devices, a speed reduction can result in regurgitant blood volume flowing from the aorta to the left ventricle, making the assessment of native left ventricular function less reliable; hence, it is important to identify the reduction in pump speed at which there is no forward or back flow (zero net flow) [7,8,20,29,41,44,49]. Another strategy is to temporarily occlude the outflow graft with a balloon which effectively prevents the back flow (i.e., regurgitant flow) during the speed turn-down [50,51].

The LVAD speed required to achieve a zero net flow varies depending on the type of the device [5,41,46,52]. George et al. performed a prospective study on the blood flow across the HeartMate II™ LVAD (Thoratec Corporation, Pleasanton, CA, USA) in patients with idiopathic dilated cardiomyopathy [46]. After ensuring an INR of ≥2.0, echocardiographic assessment of the left ventricle and peripheral hemodynamics were measured serially at three device speed settings: at a baseline device speed, 15 min after reducing the speed to 6000 rpm, and 15 min after reducing the speed to 5000 or 4000 rpm (turn-down studies). Reducing the speed to less than 6000 rpm did not have a significant effect on the left ventricular end-diastolic and end-systolic diameters, fractional shortening, or LVEF, suggesting that reducing the speed of the device to less than 6000 rpm in the assessment of the native left ventricular function is not needed. As the LVAD speed was reduced to 6000 rpm, the blood volume through the inflow cannula decreased significantly, but further speed reductions did not change the blood flow significantly, confirming that a speed less than 6000 rpm was not needed to assess the underlying left ventricular function. For the HeartWare™ LVAD (Medtronic, Minneapolis, MN, USA), the speed at which a zero net flow is likely to be achieved ranges between 1800 and 2200 rpm, while for the HeartMate 3™ LVAD (Abbott Laboratories, Chicago, IL, USA), it is between 3000 and 4300 rpm [41,51].

Serial transthoracic echocardiographic screening is necessary to identify LVAD recipients that are potential weaning candidates. In clinically stable patients, the screening can start after 2–4 weeks on LVAD support [48]. During the echocardiographic assessment on full LVAD support, the presence of the following characteristics is sought to identify the potential weaning candidates: sinus rhythm, normal or normalized LVEDD, improvement of the left ventricular wall motion, no or less than a grade 1 mitral valve or aortic valve regurgitation, a non-dilated right ventricle, and an absence or less than a grade 2 tricuspid regurgitation [7,20,41]. A progressive increase in the duration and frequency of the aortic valve opening with steady LVAD support also indicates a left ventricular functional improvement [18,49,53,54,55]. In the potential for weaning candidates, echocardiographic assessment during gradual LVAD speed reductions is recommended prior to complete cessation of the LVAD support [7,20,41,52]. If such trials elicit HF symptoms (e.g., dyspnea, chest discomfort, dizziness, or others), cardiac arrhythmias, an increase in the left ventricular dimensions beyond the normal range, or evidence of right heart instability (an increasing grade of tricuspid regurgitation or dilation of the right ventricular cavity with a reduced output), then the patient is not yet a weaning candidate and trials under ceased LVAD support should be avoided [41,42]. Pump-stop or pump turn-down studies are not indicated in patients with a prior history of stroke or transient ischemic attack, hemolysis, difficulties with anticoagulation therapy, or notably when pump thrombosis is suspected [8]. Even under optimal anticoagulation, these intervals of reduced LVAD speed should not exceed 30 min, with this time restriction raising the question of whether the information obtained during short interruptions of LVAD support can be indicative of long-term, post-explant cardiac function stability [7,20,41,42].

Inotropic reserve refers to the objective quantification of left ventricular contractility after either a pharmacologic or physiologic stress and it is reduced in patients with cardiomyopathy. Exercise stress and dobutamine stress echocardiography can provide information on the inotropic reserve and can be helpful for decision making [38,41,56]. The Harefield group reported that they performed a 6-min walk test in HeartMate II™ LVAD (Thoratec Corporation, Pleasanton, CA, USA) patients who remained asymptomatic after a 15-min pump speed reduction to 6000 rpm at rest, and they repeated their echocardiographic measurements to assess the inotropic reserve of the left ventricle [5]. In the case of the dobutamine stress test, different indices have been used to determine the inotropic reserve and the most frequently used is an absolute change in LVEF, although there is an inability to distinguish abnormalities in contractility from alterations in preload or afterload [41]. Conventionally, an absolute increase in the LVEF by 5% during a dobutamine infusion indicates a preservation of contractile reserve with a strong correlation to prognosis [57,58]. By recording the hemodynamic response during dobutamine stress echocardiography, Khan et al. evaluated 16 patients using increasing doses of dobutamine (from 5 μg/kg/min to 40 μg/kg/min) [38,59]. A hemodynamic assessment and two-dimensional echocardiography were performed at each dose level with the dobutamine stress separating the study population into two groups, namely, patients with a favorable or unfavorable (i.e., hemodynamic deterioration) response to dobutamine (9 vs. 7 out of 16 patients, respectively). The favorable dobutamine responses were characterized by an improved cardiac index, improved left ventricular force–frequency relationship (dP/dt), an improved LVEF, and a decreased LVEDD. All nine favorable responders underwent LVAD explantation, and six survived for more than 12 months.

After months of continuous unloading, even short periods of left ventricular hemodynamic loading can represent a serious challenge for an incompletely recovered left ventricle. It has been shown that recovered hearts are initially vulnerable to hemodynamic stress, with this effect diminishing over time [60]. Patients electively weaned from their LVADs by the Berlin group, underwent echocardiographic assessment of recovery at rest only and despite a lack of information on inotropic reserve and cardiac adaptation to stress, the weaning results were comparable to patient series with the additional use of dobutamine stress echocardiography and/or exercise testing [7,29,30,38,41,45,52,59]. Additional studies are therefore required to determine the benefits and potential adverse effects of myocardial overstressing during stress echocardiography for the assessment of LVAD-mediated cardiac recovery [42].

The echocardiographic assessment for recovery should be as comprehensive as possible. Table 3 includes the most useful measurements to assess cardiac improvement [42]. An accurate assessment of the mitral valve regurgitation at a reduced LVAD speed (zero net flow) is crucial. A more than moderate degree of mitral regurgitation might lead to an overestimation of the LVEF, while significant mitral regurgitation after potential device weaning might lead to a progressive deterioration of cardiac function. The Simpson’s method is usually the best for estimating the LVEF (if obtainable); although, all LVEF measurements should be taken into consideration [41]. The echocardiographic assessment should include tissue Doppler imaging (TDI) and speckle-tracking strain echocardiography (STE) [7,8,29,42,61]. Both these modalities provide important information on the native heart function improvement. The advantages of STE are that it differentiates between the active and passive movement of the ventricular wall segments, its measurements are angle-independent, and it allows for the quantification of intraventricular asynchrony and dyssynergy along with the evaluation of the myocardial contractile function, such as longitudinal myocardial shortening, that cannot be visually assessed [28,62]. However, a poor image quality, particularly when the TDI and STE measurements are performed during speed reductions and not during complete pump stops, can lead to an unreliable measurement of some of the echocardiographic parameters required to assess a weaning candidate.

### 4.2. Right Heart Catheterization

RHC is the second most important modality for the assessment of LVAD-mediated cardiac recovery in patients who tolerate a flow reduction to minimal levels and exhibit echocardiographic signs of recovery [24,29,41]. It should be performed prior to the preliminary decision-making in weaning candidates, and particularly in patients with borderline echocardiographic data and/or long-standing cardiomyopathy prior to LVAD implantation [7,41]. A thorough RHC assessment is performed at a normal pump speed followed by a reassessment after 15 min at a reduced LVAD speed allowing for a zero net flow. An outflow graft angiogram may be used to verify the device’s contribution to the flow and/or regurgitant volumes at the speed at which the hemodynamics were measured [41]. In patients supported with a continuous-flow LVAD, it has been suggested that hemodynamic assessment could take place following the occlusion of the outflow cannula with an inflated balloon, allowing for complete pump stops without any retrograde blood flow through the LVAD [28,29,50,51].

The important measurements to obtain include the right atrial pressure, pulmonary artery pressures, PCWP, the left ventricular end-diastolic pressure, and cardiac output (both by the thermodilution and Fick methods). Normal or at least borderline-normal and stable off-pump hemodynamic parameters are required for a decision in favor of device explantation [29,41,42]. A resting cardiac index which remains stable during the final pre-explant off-pump trial of at least 15 min in the operating room and which does not drop significantly from the on-pump value is an essential criterion for LVAD explantation. It has been suggested that ideally it should be >2.6 L/min/m^2^, although a cutoff level of >2.4 L/min/m^2^ has also been proposed, with the most recent example being the RESTAGE-HF trial [4,29,41,42,43]. Other pre-explant requirements include an off-pump PCWP <12 mmHg and right atrial pressure < 10 mmHg [24,29]. The RHC measurements during a supine bicycle exercise test for the assessment of cardiac recovery were also performed in LVAD recipients, but the risk-to-benefit ratio of this practice in weaning candidates has not been established [40].

### 4.3. Cardiopulmonary Exercise Testing

CPET is commonly used in the assessment of HF patients. It has been shown that exercise capacity improves in advanced HF patients following MCS [63], while pre-LVAD pVO_2_ can predict survival on LVAD support [64]. CPET can also be used for the evaluation of underlying cardiac function and as an additional tool for the assessment of weaning candidates [6,41,65,66,67]. The exercise capacity of HF patients on full LVAD support as measured by the pVO_2_ is comparable to that of heart transplant recipients, even in LVAD patients without an improvement in the native left ventricular function [68]; however, reduced left ventricular support results in a lower pVO_2_ value in comparison with that attained by the same patient on full LVAD support [18]. As such, the assessment for cardiac recovery should be performed at pump settings that provide a zero net flow. During CPET at a reduced pump speed, patients are closely observed for symptoms, and measurements at rest and at peak exercise on a modified Bruce protocol are undertaken [41]. Mancini et al. performed hemodynamic and metabolic measurements both at rest and at peak exercise with an optimal and reduced LVAD support without reported complications [68].

One study revealed that the CPET-derived data have only a limited ability to reflect the cardiac function in LVAD recipients [65]. Nevertheless, a pVO_2_ > 16 mL/kg/min was successfully used as an LVAD explantation criterion [6,24]. Cardiac power output (CPO) is a novel, central hemodynamic measure that has been suggested to be a direct indicator of overall cardiac function. The CPO is calculated by the formula: mean arterial pressure × cardiac output/451. By incorporating both the pressure and flow domains of the cardiovascular system, CPO is an integrative and unique measure of the cardiac pumping capability [41]. Both resting and peak exercise CPO have been shown to be powerful predictors of prognosis and mortality in both patients with chronic HF and cardiogenic shock [69,70]. Patients with a peak CPO less than 2 Watts have a considerably higher mortality rate than patients with a peak CPO greater than 2 Watts. Jakovljevic et al. measured the CPO in continuous-flow LVAD patients at a zero net flow during CPET and concluded that CPO is a useful and predictive marker of cardiac recovery [71]; however, considering that recovered hearts are initially vulnerable to hemodynamic stress, pVO_2_ and other CPET-derived parameter measurements should be conducted with some caution [60].

Importantly, exercise capacity significantly depends on extracardiac factors. As such, a low pVO_2_ alone does not always reliably identify patients with severe hemodynamic dysfunction during exercise [24,67]. Although a CPET assessment is useful to perform, it is typically used as an adjunct rather than a critical factor in the decision-making for device explantation [4,41]. Noteworthy in the multicenter RESTAGE-HF trial and in other studies, it was not used as part of the cardiac recovery/LVAD explantation criteria [4,7,28].

### 4.4. Pre-LVAD Weaning Assessment Suggesting Stability of Cardiac Improvement

Echocardiographic and hemodynamic data collected during short-term interruptions of LVAD support allow for the identification of patients with the potential to remain free from HF recurrence for several years after LVAD weaning (Supplementary Table S1) [42]. The stability of echocardiographic parameters during and between the LVAD interruption trials performed over 2–4 weeks after a maximum left ventricular improvement should also be considered, as this further improves the identification of such patients [27,29,42]. Because the optimal duration of LVAD support for the achievement of the maximum possible improvement varies widely from patient to patient, it has been recommended to wait until there is no further cardiac improvement, rather than to adhere strictly to a set amount of time on MCS [7,27,72].

In patients with normal RHC-derived off-pump hemodynamics before device explantation, an off-pump LVEF ≥ 45% at rest showed a predictive value of 74% for ≥5 years post-explant cardiac stability [29]. In combination with either a HF duration of ≤5 years before LVAD implantation or a normal final off-pump left ventricular end-diastolic size and/or geometry or a left ventricular peak systolic wall motion velocity ≥8cm/s, the predictive value can exceed 85% [27,29]. When considering the pre-explant stability of the left ventricular size and geometry, and the LVEF after a maximum left ventricular functional improvement, as well as during the final off-pump echocardiographic trial before LVAD weaning, the predictive value of the echocardiographic assessment for ≥5 years post-explant stability can exceed 90% [29]. Off-pump hemodynamic data alone do not sufficiently predict the long-term freedom from HF recurrence after device weaning [29,73], but a combined use of echocardiographic and RHC-derived measurements can predict the post-explant stability of cardiac function in weaning candidates who appear suitable for LVAD explantation, with an exercise test and dobutamine stress echocardiography further assisting in the decision-making process [30,38,41].

Risk factors obtained from the echocardiographic assessment of weaning candidates that have been associated with a post-explant HF recurrence are shown in Supplementary Table S2 [42]. A pre-explant LVEF < 45% or an unstable LVEF ≥ 45% (i.e., LVEF alteration compared to a previously attained maximum value and/or LVEF reduction during the final off-pump trial) can predict HF recurrence during the first three years post-weaning with an accuracy of 88% and 90%, respectively [27,29]. In patients with a pre-explant LVEF ≥ 45%, but with insufficient left ventricular structural improvement or unstable left ventricular geometry during the final off-pump trial, the probability of HF recurrence within the first three years after LVAD removal can reach 89% [27,29].

## 5. Outcomes and Quality of Life after LVAD Weaning

The probability for HF recurrence during the first post-explant year can be below 15%, even in chronic, idiopathic dilated cardiomyopathy patients [7,9,20,41,45,74,75,76]. In weaned patients with non-ischemic cardiomyopathy, the probability of a 5- and 10-year freedom from HF recurrence after LVAD explantation was 67% and 47%, respectively [29]. It was also shown that weaning can be successful even after an incomplete recovery because only 9% of the weaned patients had a pre-explant off-pump LVEF of >50%. Two other studies using a specific protocol to promote recovery showed lower rates of recurrence and had probabilities of 88.9% for a four-year, and 83.3% for a three-year freedom from HF recurrence after explantation of a pulsatile or continuous-flow LVAD, respectively [5,6]; thus, although clinically relevant cardiac recovery is relatively rare, for those who can be weaned from MCS, the chances for long-term freedom from HF recurrence are good [20,24,27,45,75,77,78]. The Harefield group compared the long-term outcomes of their LVAD patients explanted for cardiac recovery to those transplanted following LVAD support, and found similar survival rates (89.9%, 73.9%, and 73.9%, and 80.4%, 78.3%, and 78.3%, in the explanted and heart transplant groups at 1, 5, and 7 years, respectively) [19]. The rate of freedom from death or heart transplantation after LVAD weaning was 89.9%, 69%, and 69%, while the same rate after excluding a non-cardiac cause of death was 89.9%, 76.7%, and 76.7% at 1, 5 and 7 years, respectively [19]. In a recently published study employing a large European registry, the mid- to long-term outcomes in 45 patients weaned from their LVADs due to cardiac recovery appear to be encouraging, with 88% of the patients surviving without a heart transplantation, LVAD reimplantation, or HF relapse at 24 months of follow-up. Furthermore, most weaned patients suffered from only mild HF symptoms (NYHA Class I-II) [25].

A significant percentage of patients weaned from MCS can achieve cardiac and physical functional capacities that are within the normal range of healthy controls [24,45]. Jakovljevic et al. assessed the functional capacity of LVAD-explanted patients that had received the Harefield protocol who were 3.3 ± 1 years after explantation and compared them to bridge-to-transplant patients on a continuous-flow LVAD, to heart transplant candidates on the waiting list, and to healthy controls [24]. The peak exercise CPO was significantly higher in the healthy controls and explanted LVAD patients compared with advanced HF patients on the transplant list, as was the pVO_2_. In the LVAD explanted group, 38% of the patients achieved a peak CPO and 69% achieved a pVO_2_ within the ranges of the healthy controls; hence, many explanted patients had cardiac and physical functional capacities comparable to the healthy controls, and significantly better than the patients on LVAD support, suggesting that their functional recovery was sustained long-term.

Quality of life, namely, the patient’s perspective on the functional effects of illness and associated therapies, is also of utmost importance. A study assessed the long-term quality of life in patients explanted from their LVADs for cardiac recovery and compared it with patients who received an LVAD as a bridge-to-transplant and patients who underwent direct heart transplantation [79]. A total of 72 patients were studied, with 14 patients being bridge-to-recovery (3.6 ± 1.9 years post-LVAD explant), 29 bridge-to-transplant (3.3 ± 2.3 years post-transplant), and 29 heart transplant recipients without prior LVAD (3.8 ± 0.6 years post-transplant). The total *36-Item Short Form Health Survey* score was higher in LVAD weaned patients compared to both the bridge-to-transplant and direct heart transplant patients, suggesting that these patients have a better quality of life.

It should be acknowledged, however, that explanted LVAD patients should still be considered HF patients and continue with guideline-directed medical therapy, while avoiding potential precipitating events that exert substantial strain to the recovered heart (e.g., pregnancy, especially in the case of peripartum cardiomyopathy, substance or alcohol abuse, etc.). LVAD-related infections, especially in the case of LVAD decommissioning due to retained parts, pose a significant challenge with the potential to compromise sustained cardiac improvement [20,26]. For patients exhibiting a deterioration of LV function post-explantation, the treatment options include pharmacologic therapy and MCS or heart transplantation for more advanced cases. The re-implantation of an LVAD is a more challenging surgical procedure due to the previous operation, while heart transplantation, although limited by the finite number of donor organs, should expand the overall lifespan of the patient [19]. Another issue that is not well addressed in the literature is the risk of life-threatening arrhythmias and sudden cardiac death and the need of an implantable cardioverter defibrillator in this patient population [80].

There are clinical scenarios where the hemodynamic and echocardiographic data may be looking promising, but that LVAD weaning should potentially be avoided. These scenarios include patients that cannot tolerate HF guideline-directed medical therapy due to autonomic or other abnormalities, elderly patients that in the event of HF recurrence will not be candidates for LVAD re-implantation or heart transplantation (due to an advanced age), patients that are high risk to resume alcohol or substance abuse, or patients that are considered high risk for HF recurrence due to lifestyle preferences.

## 6. Current and Future Opportunities for Improvement

Although the first reports of cardiac recovery with the use of MCS in advanced HF patients were published more than 25 years ago, the field of LVAD-mediated cardiac improvement is still characterized by non-homogeneity, leading to varying and inconsistent clinical findings. The absence of i. guidelines for clinical care, monitoring, and optimization of cardiac recovery in patients supported with LVADs, ii. specific criteria for weaning candidates selection, and iii. guidance for the care of patients post-weaning, has hindered the universal pursuit of MCS-mediated cardiac recovery across centers treating advanced HF patients. Henceforth, we will discuss the current challenges in the field and potential ways to address them.

### 6.1. Inconsistency in Reported Rates of LVAD-Mediated Cardiac Recovery

The overall rate of cardiac recovery leading to device weaning in the INTERMACS database has consistently been low, occurring in less than 5% of advanced HF patients receiving LVADs [33]. On the contrary, institutions who have focused on promoting cardiac recovery, prospectively monitoring the underlying cardiac function and weaning the LVAD when patients show a significant improvement, consistently report rates of LVAD weaning that are much higher than those reported in INTERMACS [4,5,6,7,22,23,27,30,31,36,37,38,39,40]. As indicated above, these higher LVAD explantation rates in many programs worldwide are consistent with the INTERMACS reports demonstrating that the percentage of LVAD patients with an improvement of LVEF > 40% are approximately 10–15% [34,35]. Among the reasons for the reported low incidence of LVAD weaning rates in the retrospective studies and registries are the following:

- Inconsistencies regarding the native heart function monitoring in MCS recipients. Usually there is no insurance reimbursement for serial echocardiographic and hemodynamic assessment unless there is a focused institutional research interest.
- Varying HF medication regimens used in LVAD-supported patients, making their adjuvant/synergistic reverse remodeling effect inconsistent.
- Variations in the duration of LVAD unloading.
- Diversity of the studied populations in terms of HF etiology, disease chronicity and extent of adverse cardiac remodeling.
- Inconsistencies in the protocols used for the assessment of LVAD weaning candidates and LVAD weaning criteria.
- Early heart transplantation after LVAD implantation, before attesting the full effect of the MCS on the cardiac structure and function.
- Many patients that achieve a significant cardiac improvement following LVAD support elect to proceed with heart transplantation (which is considered the gold standard therapy of end-stage HF) instead of LVAD weaning, which is mostly investigational. The decision to remove an LVAD is complex and is also heavily influenced by the provider perspectives and institutional protocols and experience.

As mentioned previously, the Columbia and Utah groups studied the INTERMACS database and concluded that the incidence of cardiac recovery was higher in patients receiving an LVAD as a bridge-to-recovery, highlighting the importance of actively looking for and facilitating cardiac improvement [34,35]. Although increasingly more institutions are implementing bridge-to-recovery strategies for MCS recipients, the development of a protocolized approach for the monitoring and clinical care of LVAD-supported patients and the assessment of weaning candidates is necessitated. This could lead to an expanded adoption of such approaches and allow for a more systematic study of the MCS-mediated cardiac recovery phenomenon.

### 6.2. Optimizing LVAD-Mediated Cardiac Recovery

**Figure 3:**
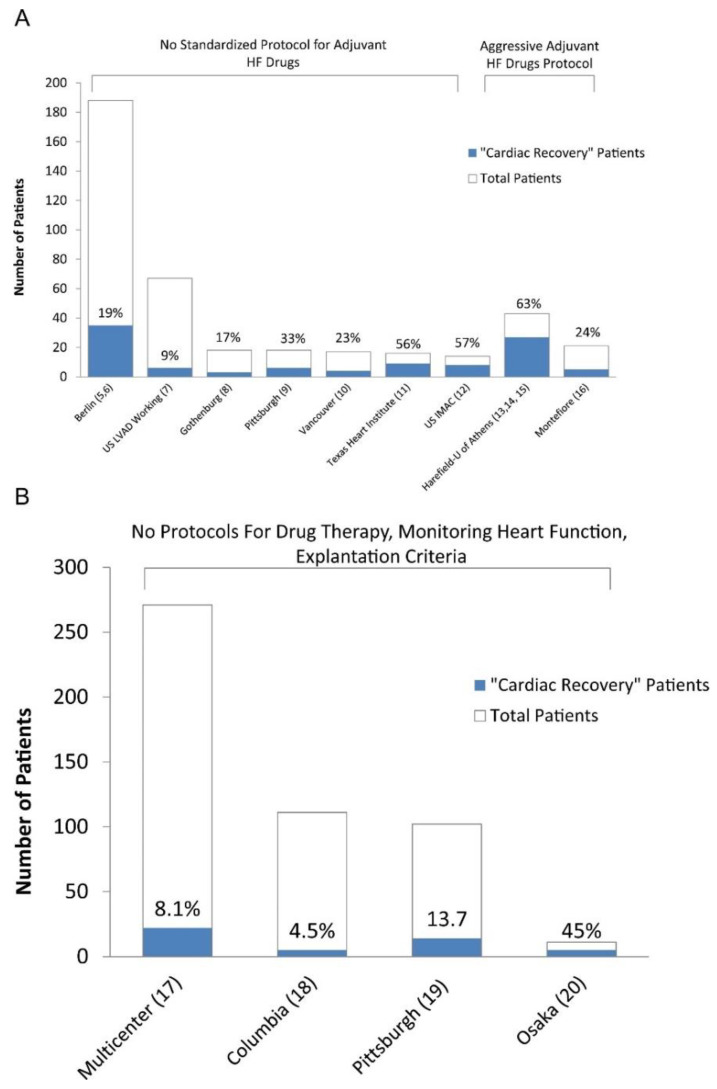
(**A**) Prospective bridge-to-recovery studies. The explantation results from the 2 studies from Harefield and the study from the University of Athens were grouped given that the same bridge-to-recovery protocol (i.e., Harefield protocol) was used in these studies. HF: heart failure; US IMAC: US Intervention in Myocarditis and Acute Cardiomyopathy. (**B**) Retrospective bridge-to-recovery studies. “Cardiac recovery” is defined as device explantation due to myocardial improvement. ***(Figure reproduced from Drakos SG et al. Clinical myocardial recovery during long-term mechanical support in advanced heart failure: Insights into moving the field forward. J Heart Lung Transplant. 2016 Apr;35[4]:413–420) [43].***

Regularly evaluating the underlying native heart function is essential to identify the LVAD-supported patients that have significantly improved their cardiac function and could be considered weaning candidates; however, the rate of cardiac recovery is also highly influenced by the attempt made to optimize recovery [41]. Usually, patients simply have their devices placed as a bridge-to-transplant or destination therapy with the adjuvant pharmacological therapies focused on blood pressure control rather than reverse cardiac remodeling. A common fallacy is that the patient has failed advanced HF medical treatment and the pump is just providing the necessary cardiac output for a patient with lifetime use or that one should simply focus on the device rather than the heart, if the patient is a heart transplant candidate [41]. Optimizing the use of LVADs as a platform to induce cardiac recovery and combining it with reverse remodeling HF medications is likely to lead to a significant increase in recovery rates. This is evident in Figure 3, in which recovery rates across published retrospective and prospective recovery studies based on the use of standardized adjuvant HF medications are depicted [43].

Mechanical unloading of the weakened left ventricle is a powerful tool to promote cardiac recovery. The LVAD directly impacts the loading conditions and forms the environment for recovery. Therefore, the LVAD speed should be serially adjusted to ensure optimal left ventricular unloading. Routine clinical practice targets a sufficient cardiac output provided by the device and echocardiographic criteria suggesting adequate left ventricular unloading, including the position of the intraventricular septum at the midline, a minimization of mitral regurgitation, and intermittent opening of the aortic valve. These recommendations, however, are not standardized and multiple studies have shown that a significant number of patients exhibit abnormal hemodynamics while on LVAD support [81,82,83,84,85]. Most of these sub-optimally unloaded patients improved their hemodynamics with an RHC-guided ramping protocol. These findings suggest that current approaches to optimize LVAD speed might be inadequate and that LVAD patients may benefit from the incorporation of an invasive hemodynamic assessment to optimize the LVAD speed and guide medical therapy. An approach that could potentially guide the hemodynamic optimization of LVAD-supported patients is the use of a pulmonary artery implantable monitor device to serially assess the hemodynamic status of patients on MCS [86]. Such an approach is currently under investigation in the INTELLECT-2 trial which recently announced very promising results (Investigation to Optimize Hemodynamic Management of Left Ventricular Assist Devices Using the CardioMEMS™ (Abbott Laboratories, Chicago, IL, USA); NCT03247829) trial [87].

The second component in optimizing cardiac recovery is the addition of adjuvant pharmacological therapy [5,6,23,41,52]. The LVAD-mediated recovery rates across the published retrospective and prospective studies grouped by the use of standardized adjuvant HF medications are depicted in Figure 2 [43]. Patients often do not tolerate neurohormonal blocking (NHB) agents while in severe HF because of renal failure or hypotension; however, LVAD support leads to an improved cardiac output, blood pressure, and renal function, with patients tolerating these agents often in high doses. The NHB agents should be initiated immediately after the weaning of inotropic support and when there is adequate end-organ recovery, then up-titrated to the highest tolerated doses. The role of newer agents that have been proven to improve the outcomes in HF patients, such as sodium-glucose cotransporter-2 inhibitors, should also be investigated in respect to their roles in promoting cardiac recovery. Recent studies have indicated a significant benefit with the use of NHB in LVAD patients, further supporting their use for promoting cardiac recovery. A recent study employing the INTERMACS database analyzed 12,144 patients and showed that those receiving any NHB agent (ACEi or angiotensin receptor blocker [ACEi/ARB], beta-blocker [BB], or mineralocorticoid receptor antagonist [MRA]) at six months after LVAD implantation (10,419; 85.8%) had a better four-year survival compared with those not receiving the NHB (56.0% vs. 43.9%, *p* < 0.001) [88]. The use of any NHB agent alone or in combination was associated with a reduced hazard of death (except for the use of MRA alone), while patients on triple NHB therapy (ACEi/ARB, BB, and MRA) exhibited the lowest hazard of death, when compared to patients not receiving the NHB (hazard ratio: 0.34, *p* < 0.001). Another important finding from this study was that a high percentage of the LVAD patients were not on an optimal guideline-directed pharmacological regimen. This indicated that there is a lot of room for improvement in the pharmacological therapy of LVAD patients. Another recent retrospective study of 307 LVAD patients across two US institutions showed that therapy with an ACEi/ARB was independently associated with a reduction of the post-LVAD mortality risk by 47% (*p* = 0.03). Other potential benefits with the use of ACEi and digoxin in LVAD patients might be a reduction in gastrointestinal bleeding [89].

Using the LVAD as a platform for adjuvant medical therapy, the RESTAGE-HF protocol resulted in high rates of cardiac recovery [4]; thus, the next steps are to focus on expanding the use of the RESTAGE-HF protocol across institutions treating LVAD recipients, and potentially increase the occurrence of LVAD-mediated cardiac recovery.

### 6.3. Predicting LVAD-Mediated Cardiac Recovery

Many institutions treating advanced HF patients do not offer a bridge-to-recovery MCS strategy as a standard therapeutic option. The allocation of the needed resources for routinely and serially assessing the native heart function of all LVAD recipients and promoting cardiac recovery is not always feasible or practical. A current unmet need in the field is the identification of patients with a high likelihood of significantly improving their cardiac structure and function upon commencing MCS. A bridge-to-recovery strategy assigned at the time of LVAD implantation could help implement diagnostic and therapeutic approaches to promote cardiac improvement and potentially wean the MCS device in those selected patients.

Retrospective studies employing registry data have identified clinical characteristics associated with a higher probability for significant reverse cardiac remodeling and LVAD weaning [25,34,35]. A younger age, shorter duration of HF, underlying non-ischemic cardiomyopathy and less dilated left ventricle have consistently been identified as the predictors of native heart function recovery on LVAD support [25,32,33,34,90]. The Utah group studied patients in the INTERMACS database and six independent predictors of LVAD weaning were identified: age < 50 years, non-ischemic HF etiology, <2 years from cardiac diagnosis, no implantable cardioverter defibrillator (likely a surrogate of a short duration of HF), a serum creatinine level < 1.2 mg/dL, and LVEDD < 6.5 cm [35]. Although the clinical characteristics typically associated with cardiac recovery can be viewed as surrogates for the potential reversibility of chronic cardiac adverse remodeling, a more direct research target would be the identification of pre-implant molecular and/or biochemical markers as the predictors of MCS-mediated cardiac improvement. Indeed, the Working Group of the National Heart, Lung and Blood Institute aiming at advancing the science of cardiac recovery with MCS, identified a dissociation between functional outcomes and the underlying biological derangements as a critical shortcoming in the field [43,91]. The correlation of the underlying biological milieu, with clinical and hemodynamic parameters serving as surrogates for cardiac structural and functional improvement, could lead to the identification of biomarkers useful to stratify patients based on their cardiac recovery potential prior to LVAD implantation. In two recently published studies, investigators examined cardiac tissue and blood obtained prior to LVAD implantation from patients who eventually improved their cardiac structure and function on MCS and from patients who did not. In the first study, the authors concluded that an intact t-tubule system at the time of LVAD implantation may constitute a precondition and predictor for cardiac recovery after MCS, while in the second it was deduced that myocardial and circulating cytokine levels correlate with the potential of the failing heart to recover after LVAD unloading [92,93].

Such efforts, leveraging the access to cardiac tissue and blood and correlating molecular data with clinical outcomes in advanced HF patients receiving MCS, will help us reach a more tailor-made level of stratifying patients based on their cardiac recovery potential and consequently better allocate resources to increase the chances of cardiac recovery and MCS weaning.

### 6.4. The Importance of Promoting “Partial” Cardiac Recovery

Cardiac recovery after LVAD-facilitated volume and pressure unloading is not an “all or none” phenomenon (such as pregnancy or death) but rather it manifests in a continuous spectrum [32,43]. Although a relatively small proportion of advanced HF patients may have a complete normalization of cardiac function, a much larger proportion of patients experience a “partial” cardiac recovery that renders them similar to stable ambulatory HF patients [43]. A recent multicenter study of 358 consecutive advanced HF patients with a reduced LVEF showed that 34 (10%) patients achieved an LVEF ≥ 40% and an LVEDD ≤ 6.0 cm at the last available echocardiographic follow-up timepoint within one year of LVAD support and were termed “responders”, while an additional 112 (31%) patients had an absolute LVEF improvement of ≥5% and were termed “partial responders” [32]. This partial responder group had a median increase of LVEF equal to 9% (range 6–14%).

Reverse cardiac remodeling in HF patients with a reduced LVEF treated with NHB and cardiac resynchronization therapy is associated with improved survival [94,95,96]. Even a 5% improvement in LVEF corresponds to a 14% reduction in mortality, suggesting that improvements in LVEF with a therapeutic intervention may serve as a surrogate for clinical outcomes [97]. The concept of grading the reverse cardiac remodeling by absolute changes in LVEF in HF patients is well established; however, this has not been applied to LVAD-supported patients. Whether an LVAD patient classified as a “partial responder” (and “responder”) has improved clinical outcomes while staying on mechanical circulatory support, as has been demonstrated in patients with HF and a reduced LVEF, will have to be validated in future prospective studies [32]. Along these lines, a recent single-center study from Duke University suggested that cardiac improvement following LVAD support is associated with favorable clinical outcomes and side effect profiles [98,99]. “Partial” cardiac recovery might also be worth pursuing, as an improved native left ventricular contractility may improve the exercise tolerance and functional capacity in patients on LVAD support [66,100,101,102], and prevent or reverse the abnormal physiologic sequalae attributed to an absence of arterial pulsatility in patients with continuous-flow LVADs [103,104,105,106]. Furthermore, cardiac recovery promotion could potentially induce right ventricular reverse remodeling, leading to a reduction in right ventricular failure and an improvement of the overall hemodynamic status of patients supported with MCS [102,107]. Furthermore, such “partial responder” patients may benefit from optimization of their LVAD speed/mechanical unloading and/or titration of guideline-directed HF medications that could lead to further improvement of their left ventricular structure and function, and ultimately become “responders”.

### 6.5. Predicting and Improving the Sustainability of LVAD-Mediated Cardiac Recovery

Although the long-term outcomes after MCS weaning due to cardiac recovery have been encouraging, as most weaned patients remain free from recurrent HF, reimplantation of an LVAD, or heart transplantation, the sustainability of cardiac recovery remains a point of concern for physicians treating advanced HF patients. As discussed above, there are echocardiographic and hemodynamic data suggestive of sustained heart function improvement; however, our knowledge of the clinical and molecular markers of stable cardiac recovery could be improved.

A potential key piece that is currently missing from the field of MCS-mediated cardiac recovery is a uniform utilization of the LVAD as a cardiac therapy [41]. The way the left ventricle gets unloaded, and, importantly for the concept of cardiac recovery, how it gets reloaded, remains undefined. Intermittently decreasing the LVAD speed to allow an increased ventricular preload, sufficient contractility to open the aortic valve, and increased myocardial work, might be a way to retrain and rehabilitate the left ventricle. Patients may benefit from defined periods of significantly reduced LVAD support, and this concept has already been in place, to some degree, with the Jarvik 2000™ LVAD (Jarvik Heart, Inc., New York, NY, USA) and its intermittent low speed technology. In published reports, this feature has been used to gradually reload the left ventricle over several months to allow for reconditioning prior to LVAD explantation [26,108,109]. Cardiac metabolism data derived from LVAD patients have also provided indirect support for such a controlled reloading and conditioning strategy [110].

Whether the use of MCS as a cardiac reconditioning therapy could help enhance the rates of sustained LVAD-mediated cardiac recovery remains to be investigated using a standardized left ventricular reloading approach in a prospective setting.

### 6.6. Improving Left Ventricular Assist Devices

The field of durable MCS has enjoyed significant technological advances during the last decade, and great strides have been made to improve the clinical outcomes for LVAD recipients [111,112]. Adverse events such as stroke, infection, gastrointestinal bleeding, and right heart failure, remain the Achilles’ heel of MCS, with a major contribution to morbidity and mortality [113,114,115]. Furthermore, the burden of adverse events might be driving physicians and patients away from the option of MCS and instead towards the alternative treatment routes of heart transplantation or palliative care, not giving the opportunity to appropriately selected patients with advanced HF to explore the possibility of an LVAD-mediated cardiac recovery. Moreover, we are currently unaware of possible implications that adverse events may have on the potential for LVAD-mediated cardiac improvement through the activation of adverse cardiac remodeling pathways.

It is imperative that all involved stakeholders in healthcare and industry maximize their efforts for further improving LVAD technology to minimize adverse events and incorporate modalities that might be beneficial for cardiac recovery. Such approaches include the elimination of external cables, a major source of infection, and the establishment of wireless charging [116,117]; the use of biocompatible surfaces to reduce the need for anticoagulation and attenuate hemocompatibility-related adverse events [117]; the development of automated control strategies based on the left ventricular afterload impedance to allow the optimization of unloading and a controlled reconditioning of the left ventricle [118]; and the incorporation of pulsatility, as older generation, pulsatile-flow devices, have been associated with a higher probability for cardiac recovery compared to the newer generation, continuous-flow devices [103,104,106,119].

### 6.7. LVADs as a Platform to Advance the Science of Reverse Remodeling and Myocardial Recovery

Understanding the mechanisms of cardiac recovery following MCS may be paramount to understand and facilitate cardiac improvement in the broader HF population. An existing gap of knowledge in the field of MCS-mediated cardiac recovery is the correlation of functional outcomes with molecular, cellular, and histological findings [91]. It is currently under intensive investigation which of the observed biological changes during MCS are associated with true cardiac recovery mechanisms versus epiphenomena. Combined functional and biological studies, by examining paired tissue samples at the time of LVAD implantation and explantation (due to cardiac recovery or heart transplantation, etc.) from patients with various degrees of LVAD-mediated cardiac improvement, could help distinguish between the true mechanistic insights as opposed to mere epiphenomena. In-depth investigational efforts incorporating a rigorous basic science approach could assign causality and lead to the discovery of novel therapeutic targets [91,92,120,121,122,123]. Meanwhile, extrapolating the lessons learned from the LVAD investigational setting could be transformational for the greater HF population. The overarching goal is to improve our understanding of cardiac biology and the associated molecular, cellular, and structural signatures of cardiac recovery, and to manipulate them in a way that could be beneficial for the greater HF population [123].

## 7. Conclusions

With an aging population and a finite number of donor organs available for transplantation, it is likely that more patients at the higher end of HF severity will be undergoing treatment with an MCS device in the future. At the same time, a gradual shift towards destination therapy as the indication for implantation observed in recent years will probably lead to more patients being supported with LVADs without having heart transplantation as an exit treatment strategy. In such a setting, it is crucial that MCS recipients, and especially those with a higher potential for cardiac improvement, are actively followed and optimized for cardiac recovery. Potential LVAD weaning allows the patients to live with their native hearts and attain a good quality of life, while avoiding heart transplantation and sparing donor hearts for other individuals in need. Even if LVAD-weaned patients should decompensate and require transplantation at a later stage, this approach is likely to extend their overall life span.

Standardized monitoring and therapeutic protocols to facilitate cardiac recovery on LVAD support, specific LVAD weaning evaluation and explant-decision criteria are warranted to allow for the institution of a standard bridge-to-recovery approach. Patients who “partially” recover should be further optimized medically and hemodynamically to potentially further improve their cardiac structure and function, while patients who achieve a significant cardiac improvement should undergo LVAD weaning evaluation, and eventual LVAD weaning should be pursued. The accurate prediction of patients with a high propensity for cardiac improvement on MCS and sustained cardiac recovery following LVAD weaning, will allow for a better allocation of patients to the optimal, advanced HF treatment option. Innovation in the field of MCS devices is also warranted, to improve patient outcomes and instill confidence in the use of LVAD therapy, to increase the pool of advanced HF patients undergoing the route of MCS to explore the possibility of MCS-mediated cardiac recovery, and to incorporate device modalities to potentially enhance cardiac improvement. Lastly, capitalizing on the MCS investigational setting to improve our understanding of the biological mechanisms driving HF and cardiac recovery could be transformational for the diagnostics and therapeutics applicable to the greater HF population.

## Figures and Tables

**Table 1:** Prospective Studies Investigating Cardiac Functional and Structural Improvement during Chronic LVAD Support.

Group, Year	No. of Patients	HF Etiology	Standardized Pharmacologic Therapy	Heart Function Monitoring Protocol	LVAD Support Duration (months)	Cardiac Recovery *	Freedom from LVAD Reimplantation or HTx, Follow-Up Duration
Pittsburgh, 2003 [37]	18	NICM: 72% ICM: 28%	No	Yes	8	NICM: 38% ICM: 20%	67%, 16.5 months
Texas Heart Institute, 2003 [38]	16	NICM: 75% ICM: 25%	Yes	Yes	8	NICM: 58% ICM: 50%	78%, 14.3 months
Gothenburg, 2006 [40]	18	NICM: 83% ICM: 17%	No	Yes	7	NICM: 17% ICM: 0%	33%, 8 years
Harefield, 2006 [6]	15	NICM: 100%	Yes	Yes	11	NICM: 73%	100% and 89%, 1 and 4 years, respectively
U.S. LVAD Working Group, 2007 [30]	67	NICM: 55% ICM: 45%	No	Yes	4.5	NICM: 13.5% ICM: 3.3%	100%, 6 months
University of Athens, 2007 [22]	8	NICM: 100%	Yes	Yes	7	NICM: 50%	100%, 2 years
Berlin, 2008 and 2010 [7,27]	188	NICM: 100%	No	Yes	4	NICM: 19%	74% and 66%, 3 and 5 years, respectively
Vancouver, 2011 [39]	17	Not reported	No	Yes	7	NICM and ICM: 23%	100%, 2 years
Harefield, 2011 [5]	20	NICM: 100%	Yes	Yes	9	NICM: 60%	83%, 3 years
U.S. IMAC, 2012 [36]	14	NICM: 100%	No	Yes	3.5	NICM: 67%	87.5%, 17.5 months
Montefiore, 2013 [23]	21	NICM: 62% ICM: 38%	Yes	Yes	9	NICM: 23% ICM: 0%	100%, 57 months
Utah Cardiac Recovery Program, 2016 [31]	154	NICM: 60% ICM: 40%	No	Yes	6	NICM: 21% ICM: 5%	N/A
RESTAGE-HF Multicenter Trial, 2020 [4]	40	NICM: 100%	Yes	Yes	13	NICM: 48%	90% and 77%, 1 and 3 years, respectively

* Cardiac recovery was defined in all studies (except the Utah Cardiac Recovery study) as LVAD explantation due to cardiac functional and structural improvement (however, the degree of improvement and specific criteria varied between studies). In the Utah Cardiac Recovery study, cardiac recovery was defined as post-LVAD left ventricular ejection fraction ≥ 40% in at least 2 consecutive turn-down echocardiograms and no drop in LVEF < 40% at a later timepoint (independently of whether the device was eventually explanted). HF: heart failure; HTx: heart transplantation; ICM: ischemic cardiomyopathy; NICM: nonischemic cardiomyopathy; LVAD: left ventricular assist-device; N/A: not applicable.

**Table 2:** Assessment of LVAD-supported patients for cardiac structural and functional cardiac improvement and potential device weaning. ***(Table reproduced from Drakos SG et al. Clinical myocardial recovery during long-term mechanical support in advanced heart failure: Insights into moving the field forward. J Heart Lung Transplant. 2016 Apr;35[4]:413–420) [43].***

Parameters and Parameter-Derived Measurements During Ore-Explant Off-Pump Trials (At Rest, without Inotropic Myocardial Support)
**Stage 1—Screening phase: serial cardiac structural and functional evaluation (suggested duration 6–12 months)** Serial echocardiography ○ Monthly or bimonthly ○ Full LVAD support and minimal LVAD support for 15–30 min Patients revealing favorable findings (e.g., LVEF > 40–45% and LVEDD < 60 mm) proceed to Stage 2 **Stage 2—Weaning phase** Exercise capacity testing and hemodynamic evaluation ○ Right heart catheterization: full and minimal LVAD support for 15–30 min ○ Exercise capacity and myocardial reserve (6-min walk test or cardiopulmonary exercise test or dobutamine stress test): minimal LVAD support LVAD weaning criteria: structure, function, and hemodynamics (values at minimal LVAD support and/or peak exercise) ○ Echocardiogram ▪ LVEDD < 60 mm ▪ LVESD < 50 mm ▪ LVEF > 45% ○ Right heart catheterization ▪ PCWP < 15 mm Hg ▪ CI > 2.4 L/min/m^2^ ○ Cardiopulmonary exercise test ▪ VO_2_ max > 16 mL/kg/min ▪ VE/VCO2 < 40

CI: cardiac index; LVEDD: left ventricular end diastolic diameter; LVEF: left ventricular ejection fraction; LVESD: left ventricular end systolic diameter; PCWP: pulmonary capillary wedge pressure; VE/VCO_2_: slope of ventilation versus carbon dioxide production; VO_2_ max: maximal oxygen consumption; LVAD: left ventricular assist device.

**Table 3:** Echocardiographic measurements and measurement-derived parameters for evaluation of cardiac recovery during off-pump/pump turn-down trials. Reprinted with permission from Ref. [42]. Copyright 2019 Elsevier.

ECHO Techniques	Measurements and Key Parameters
M-Mode and 2D ECHO	Left ventricle (LV) ○ End-diastolic diameter (LVEDD) in the PLAX view ○ End-systolic diameter (LVESD) in the PLAX view ○ End-diastolic relative wall thickness (RWT_ED_) ∗ in the PSLAX view ○ End-diastolic short/long axis ratio (S/L_ED_) in the apical 4C view ○ Ejection fraction (LVEF) Right ventricle (RV) ○ End-diastolic dimensions (on parasternal and apical views) ○ End-diastolic short/long axis ratio (S/L_ED_) ○ Fractional area change (FAC) ○ Tricuspid annulus peak systolic excursion (TAPSE)
Flow-Doppler imaging (CW-Doppler, PW-Doppler color-flow mapping)	Parameters and indices of LV diastolic function (apical 4C views) ○ Transmitral flow E and A wave velocity, E wave deceleration time, E/A velocity ratio ○ Isovolumetric relaxation time Parameters for LV systolic function ○ Isovolumetric contraction time (apical 4C view) ○ Stroke volume (SV) ^†^ Detection and quantification of cardiac valve regurgitations Pulmonary arterial systolic pressure estimation (apical 4C view, in patients with TR)
Tissue-Doppler imaging	LV systolic wall motion peak velocity (Sm) (measured with PW-TD at the basal posterior wall on parasternal view images) Tricuspid lateral annulus peak systolic wall motion velocity (TAPS’) (measured with PW-TD on apical 4C view images)
Speckle tracking 2D-strain imaging	LV radial, circumferential and longitudinal global peak systolic strain and strain rate LV intraventricular dyssynchrony index of contraction (IVDSI_LV_) ^‡^ LV dyssynergy index of contraction ^§^

2D: 2-dimensional; A wave: late filling velocity (atrial contraction); CW: continuous wave; ECHO: echocardiography; E wave: early filling velocity; 4C: 4 chamber; PW: pulsed wave; PW-TD: pulsed-wave tissue Doppler; PLAX; parasternal long axis; TR: tricuspid valve regurgitation. ∗ RWT_ED_ = (end-diastolic interventricular septum thickness + end-diastolic posterior wall thickness)/LVEDD. ^†^ SV = LV outflow tract (LVOT) cross-section area (in the PSLAX view). Velocity time integral obtained by tracing the PW-Doppler signal’s envelope in the LVOT (measured on apical view images). ^‡^ IVDI_LV_ = standard deviation (SD) of the time-to-peak systolic strain (TPS)/mean value (M) of time-to-peak systolic strain (IVDI_LV_ = SD_TPS_/M_TPS_). ^§^ LV dyssynergy index = coefficient of variance of the 6 regional strain values at the end of the LV systole, before the aortic valve closure, and/or at the LV mid-systole.

## Supplementary Materials

The following supporting information can be downloaded at: https://www.mdpi.com/article/10.3390/jcm11123542/s1, Table S1. Clinical value of echocardiography and RHC for pre-explant prediction of long-term freedom from HF recurrence in case of LVAD explantation. Table S2. Clinical value of echocardiography for pre-explant risk assessment for HF recurrence after LVAD explantation.
