# Supplementary material for: LVAD as a Bridge to Remission from Advanced Heart Failure: Current Data and Opportunities for Improvement
Source: J Clin Med. 2022 Jun 20;11(12):3542. doi: 10.3390/jcm11123542 (PMC9225013; doi:10.3390/jcm11123542)
Supplement: Supplementary file 1 [file jcm-11-03542-s001.zip › jcm-1751755-supplementary.pdf]

Supplemental Table S1. Clinical value of echocardiography and RHC for pre-explant prediction of long-term freedom from HF recurrence in case of LVAD explantation. ***(Table reproduced from Dandel M, et al. Evaluation of Cardiac Recovery in Ventricular Assist Device Recipients: Particularities, Reliability, and Practical Challenges. Can J Cardiol. 2019 Apr;35(4):523-534) (42)***

Examination	Parameters and parameter-derived measurements during pre-explant off-pump trials (at rest, without inotropic myocardial support)	Predictive value for ≥ 5 years postweaning cardiac stability
TTE in patients with off-pump RHC measurements in the normal range	LVEF ≥ 45% during the last pre-explant TTE off-pump∗ trial	74%†
	LVEF ≥ 45% plus normal LVEDD (≤ 55 mm at BSA ≤ 1.8 m^2) during the last pre-explant TTE off-pump∗ trial	86%†
	LVEF ≥ 45% plus RWTLV ≥ 0.38	87%†
	Stable pre-explant LVEF ≥ 45% after maximal LV improvement and during the last pre-explant TTE off-pump∗ trial	86%†
	LVEF ≥ 45% plus Sm ≥ 8cm/s, stable after maximum improvement and during the last pre-explant TTE off-pump∗ trial	87%†
	Stable pre-explant LVEF ≥ 45% plus stable normal LVEDD after maximal LV improvement and during the last pre-explant TTE off-pump∗ trial	94%†
	Stable VTI in the LVOT (PW-Doppler) during the last pre-explant TTE off-pump∗ trial (ie, stable LV stroke volume)<br>No or only mild MR and/or AR<br>No RV dilation (end-diastolic RVOT diameter < 35 mm, RV short/long axis-ratio in the apical 4C view < 0.6)<br>No or less than grade II tricuspid and/or pulmonary valve regurgitation	Alone not predictive for long-term post-explant freedom from HF recurrence, but all these Doppler-derived measurements, plus the exclusion of RV dilation, are a precondition for weaning success
RHC‡	CI > 2.6L/min/m^2 BSA, without continuous reduction up to the end of the final pre-explant off-pump trial<br>PCWP < 13 mm Hg, without continuous increase up to the end of the final pre-explant off-pump trial<br>Right atrial pressure (mean) < 10 mm Hg without continuous increase up to the end of the final off-pump trial	RHC data are alone not predictive for long-term post-explant freedom from HF recurrence, but are a precondition for successful weaning

BSA: body surface area; CI: cardiac index; 4C: 4 chamber; HF: heart failure; LV: left ventricle; LVAD: left ventricular assist device; MR and AR: mitral regurgitation and aortic regurgitation, respectively; LVEF: left ventricle ejection fraction; LVEDD: left ventricular end-diastolic diameter; LVOT: LV outflow tract; PCWP: pulmonary capillary wedge pressure; PW: pulsed wave; RWTLV: left ventricular relative wall thickness ([posterior wall thickness + ventricular septum thickness]/LVEDD); RHC: right heart catheterization; TTE: transthoracic echocardiography; VTI: velocity-time integral.
∗ Off-pump measurements imply either temporary pump stops (true off-pump trial) or temporary pump turn-down to values resulting in ± zero flow in 1 cardiac cycle, depending on whether the LVAD is a pulsatile- or a continuous-flow pump.
† Predictive value of the TTE parameter for ≥ 5years freedom from HF recurrence is valid only for patients with normal RHC-derived off-pump hemodynamic measurements before explantation.
‡ Measurements obtained during repeated pump stops with balloon occlusion of the outflow graft in CF LVAD recipients.

Supplemental Table S2. Clinical value of echocardiography for pre-explant risk assessment for HF recurrence after LVAD explantation. ***(Table reproduced from Dandel M, et al. Evaluation of Cardiac Recovery in Ventricular Assist Device Recipients: Particularities, Reliability, and Practical Challenges. Can J Cardiol. 2019 Apr;35(4):523-534) (42)***

ECHO approach	Parameters and parameter-derived measurements during pre-explant off-pump trials in LVAD recipients with no worse than borderline RHC-derived off-pump hemodynamic measurements	Predictive value for HF recurrence after ≤ 3 years
Standard TTE at rest during interruption of LVAD support	LVEF < 45% during the last pre-explant off-pump trial	88%
	LVEF < 45% during the last pre-explant off-pump trial plus pre-implant HF duration of > 5 years	≈ 100%
	Unstable LVEF ≥ 45% with pre-explant alteration of > 10% of best value	90%
	LVEF ≥ 45% without normalization of LV size (insufficient reverse remodelling with LVEDD > 55 mm at BSA ≤ 1.8 cm2)	89%
	LVEF ≥ 45% with insufficient reverse remodelling (RWTLV < 0.38)	82%
	LVEF ≥ 45% with unstable reverse remodelling (RWTLV reduction of > 8% during the last pre-explant off-pump trial)	87%
	LVEF ≥ 45% with unstable LV geometry (S/LED increase of > 10% during the last pre-explant off-pump trial)	85%
	LVEF ≥ 45% in the presence of low systemic APd (≤ 50 mm Hg)	Proven risk factors no data on their predictive value
	Transmitral restrictive flow-profile (accentuation or new appearance)	
	VTI reduction in the LVOT (= stroke volume reduction) during the final off-pump trials	
	TR (new appearance or accentuation) and/or TR jet velocity increase during the final off-pump trials	
	RVEDD increase ± RV geometry alterations during the final off-pump trial	
TDI & 2D-STE at rest during interruption of LVAD support	LVEF ≥ 45% with low wall-motion velocity (Sm < 8 cm/s)	83%
	LVEF ≥ 45% with unstable wall-motion velocity (Sm alteration of > 10% during the final off-pump trials)	90%
	LV myocardial asynchrony and dyssynergy during the final off-pump trials	Risk factor; no data on predictive value
Off-pump DSE	Minor LVEF increase during DSE (absolute increase in LVEF by < 5%)	Risk factor; no data on predictive value
TTE after 6 MW	LVEF < 50% after 6MW	Risk factor; no data on predictive value

APd: diastolic arterial pressure; BSA: body surface area; DSE: dobutamine stress echocardiography; ECHO: echocardiography; HF: heart failure; LV: left ventricle; LVAD: left ventricular assist device; LVEF: left ventricular ejection fraction; LVOT: left ventricular outflow tract; RHC: right heart catheterization; RV: right ventricle; RVEDD: RV end-diastolic diameter; RWTLV: LV end-diastolic relative wall thickness; S/LED: LV end-diastolic short/long axis ratio; 6MW: 6-minute walk; Sm: systolic wall motion peak velocity at the basal LV posterior wall; TR: tricuspid regurgitation; TDI: tissue Doppler imaging; TTE: transthoracic echocardiography; 2D-STE: 2-dimensional speckle tracking-derived echocardiography; VTI: velocity-time integral.
